# Peripheral Blood miR-328 Expression as a Potential Biomarker for the Early Diagnosis of NSCLC

**DOI:** 10.3390/ijms140510332

**Published:** 2013-05-16

**Authors:** Paola Ulivi, Giovanni Foschi, Marta Mengozzi, Emanuela Scarpi, Rosella Silvestrini, Dino Amadori, Wainer Zoli

**Affiliations:** 1 Biosciences Laboratory, Istituto Scientifico Romagnolo per lo Studio e la Cura dei Tumori (IRST), IRCCS, 47014 Meldola, Italy; E-Mails: giova.foschi@gmail.com (G.F.); rosella.silvestrini@istitutotumori.mi.it (R.S.); w.zoli@irst.emr.it (W.Z.); 2 General Thoracic Surgery, Department of Thoracic Diseases, Morgagni-Pierantoni Hospital, 47121 Forlì, Italy; E-Mail: m.mengozzi@ausl.fo.it; 3 Unit of Biostatistics and Clinical Trials, Istituto Scientifico Romagnolo per lo Studio e la Cura dei Tumori (IRST), IRCCS, 47014 Meldola, Italy; E-Mail: e.scarpi@irst.emr.it; 4 Department of Medical Oncology, Istituto Scientifico Romagnolo per lo Studio e la Cura dei Tumori (IRST), IRCCS, 47014 Meldola, Italy; E-Mail: direzione.scientifica@irst.emr.it

**Keywords:** non small cell lung cancer, miRNAs, diagnosis, serum

## Abstract

Lung cancer is often diagnosed at an advanced stage, with subsequently poor prognosis. There are no biomarkers available to facilitate early diagnosis or to discriminate between benign and malignant nodules. MicroRNAs (miRNAs) are stable molecules that can be found and measured in peripheral blood, thus representing potential diagnostic biomarkers. We evaluated 100 individuals comprising 86 patients with predominantly early-stage non-small cell lung cancer (NSCLC) and 24 healthy donors. RNA was extracted from peripheral blood samples and the expression of a panel of miRNAs was analyzed by Real-Time PCR method. Expression levels of miR-328, miR-18a, miR-339 and miR-140 were significantly higher in NSCLC patients than in healthy donors (*p* < 0.05). In particular, miR-328 showed good diagnostic accuracy in discriminating between patients with early NSCLC and healthy donors (AUC ROC 0.82, 95% CI 0.72–0.92), with 70% sensitivity and 83% specificity at the best relative expression cut-off of 300. Moreover, miR-339 was a good discriminant between healthy donors and late-stage NSCLC patients (AUC ROC 0.79, 95% CI 0.68–0.91). In conclusion, miR-328 represents a potential diagnostic biomarker of NSCLC, especially for the identification of early-stage tumors. Its role in discriminating between benign and malignant nodules detected by spiral CT warrants further investigation.

## 1. Introduction

Lung cancer is the leading cause of cancer death worldwide and five-year survival is closely correlated with tumor stage at the time of diagnosis. Early detection could therefore represent a promising strategy to increase curability and reduce mortality. A 20% reduction in mortality has been reported with the use of low-dose spiral computed tomography (CT) in high-risk individuals [1]. However, the radiation doses delivered and high costs limit the widespread application of this technique as a screening procedure [2]. Moreover, the high rate of false-positives means that a large proportion of individuals undergo unnecessary follow-up and other diagnostic tests, including biopsy, further increasing costs and health risks [1–3]. Several biomarkers have been proposed to facilitate early diagnosis or to monitor suspicious nodules detected by spiral CT.

microRNAs (miRNAs) are small non-coding RNAs that play a critical role in both physiologic and pathologic processes, such as cancers [4–7]. In addition, miRNAs in human serum or plasma have been shown to have much stronger stability than high molecular weight RNA [8–10] due to their resistance to RNase digestion. These findings make miRNA a potentially important tool for cancer diagnosis using blood samples.

Previous studies have revealed a deregulation of miRNA expression in the blood cells of cancer patients compared to those of healthy individuals [11–15], suggesting that miRNA could represent a potential biomarker for detecting malignancies. Moreover, it has been seen that a specific miRNA profile is capable of distinguishing between NSCLC and chronic obstructive pulmonary disease [16].

In the present study, we analyzed expression levels of a panel of miRNAs extracted from the peripheral blood of NSCLC patients and healthy donors in order to identify specific miRNAs that can be used as cancer biomarkers or to monitor nodules detected by spiral CT.

## 2. Results and Discussion

### 2.1. Identification of the Best Housekeeping miRNAs

The best housekeeping miRNAs to analyze in peripheral blood were selected by evaluating the threshold cycle (Ct) values of RNU6B, RNU38B, RNU58A, RNU43 and RNU49 in 20 NSCLC patients and 20 healthy donors. Ct values were then inserted into geNorm software which identified RNU38B, RNU58A and RNU43 as the most stable housekeeping miRNAs. These three miRNAs were analyzed in the entire case series of 86 NSCLC patients and 24 healthy donors, and the geNorm analysis subsequently performed on the results revealed M values of 1,033, 1,047 and 15,023 for RNU38B, RNU58A and RNU43, respectively. Consequently, only the first two housekeeping miRNAs were considered for the final analysis.

### 2.2. Expression Levels of miRNAs

We first analyzed expression levels of the following starting panel of miRNAs in 17 healthy donors and 23 NSCLC patients: miR-1224, miR-155, miR-21, miR-22, miR-328, miR-423, miR-93, let-7, miR-126, miR-140, miR-18a, miR-19a, miR-339 and miR-361. Preliminary results highlighted the following six miRNAs whose median expression differed between the healthy donors and NSCLC patients: miR-328, miR-423, miR-140, miR-18a, miR-339 and miR-361 (*p =* 0.08, 0.08, 0.08, 0.02, 0.003 and 0.02, respectively). These six miRNAs were then analyzed in the entire case series of 24 healthy donors and 86 NSCLC patients.

**Figure 1:**
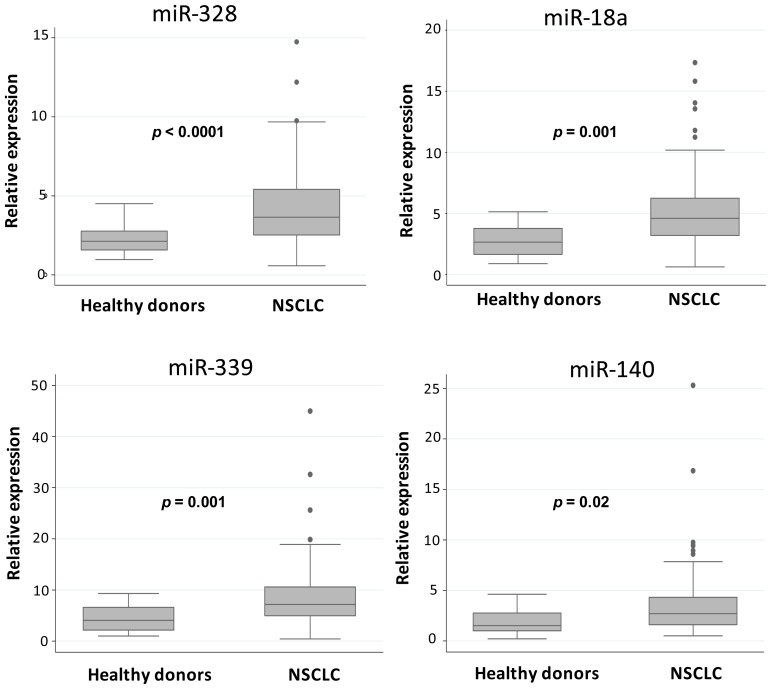

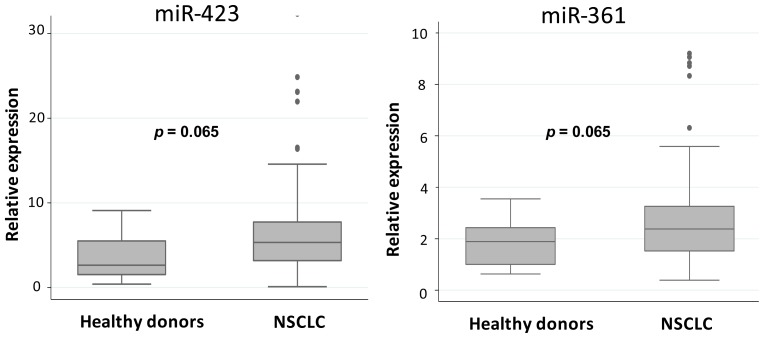
Box plot representation of miR-328, miR-18a, miR-339, miR-140, miR-423 and miR-361 in healthy donors and NSCLC patients.

Expression levels of miR-328, miR-18a, miR-339 and miR-140 were significantly higher (*p* < 0.0001, *p* = 0.001, *p* = 0.001, *p* = 0.021, respectively) in NSCLC patients than in healthy donors. miR-423 and miR-361 expression was higher, albeit not significantly, in cancer patients compared to controls (*p* = 0.065) (Figure 1).

No significant correlations were found between expression levels of any of the six miRNAs and age, gender, or smoking habits in healthy donors. In NSCLC patients, expression levels of miR-18a were significantly lower in smokers compared to never or former smokers (*p* = 0.03). Moreover, miR-423 expression was significantly higher in SCC compared to ADC tumors (*p* = 0.013), and miR-140 and miR-361 expression levels were higher, albeit not significantly, in advanced-stage (III and IV) with respect to early-stage (I and II) tumors (*p* = 0.07).

### 2.3. Diagnostic Potential of miRNAs

**Figure 2:**
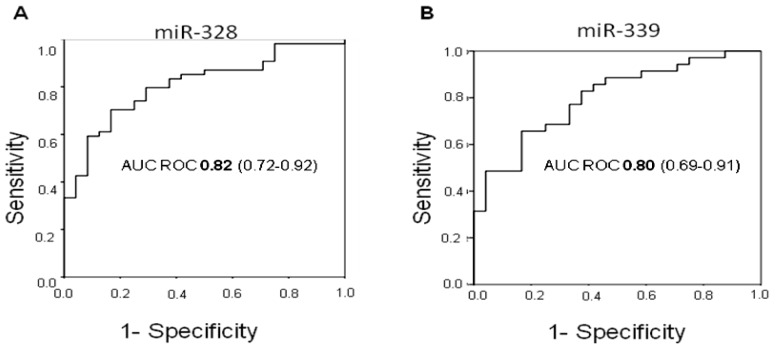
(**A**) ROC curve of miR-328 showing discrimination between healthy donors and early-stage tumors; (**B**) ROC curve of miR-339 showing discrimination between healthy donors and late-stage tumors.

The diagnostic relevance of each miRNA, singly or in combination, was also analyzed. The area under the ROC curve (AUC) (Table 1) indicated that miR-328 was the best diagnostic discriminant in the overall case series (AUC ROC 0.79, 95% CI 0.70–0.89) and this was further confirmed when the group of early-stage tumors was considered (AUC ROC 0.82, 95% CI 0.72–0.92) (Figure 2A).

Sensitivity varied from 33% to 82% and specificity from 67% to 100% at the different cutoffs considered (Table 2).

Diagnostic accuracy was not influenced by age, gender or smoking habits (data not shown). The relation between miR-328 expression and risk of early-stage lung cancer, analyzed by the fitted logistic regression model, showed an odds ratio (OR) of 2.86 (95% CI 1.62–5.05, *p* < 0.0001). Conversely, miR-339 proved to be the best marker to discriminate between healthy donors and late-stage tumors (AUC ROC 0.80, 95% CI 0.69–0.91) (Figure 2B).

### 2.4. Combined Analysis of miRNAs

**Figure 3:**
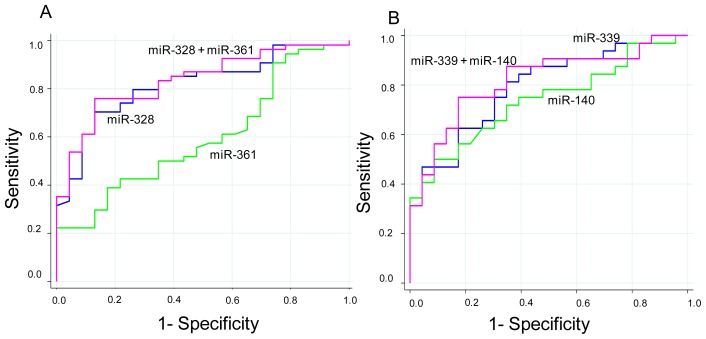
(**A**) ROC curve of miR-328 in combination with miR-361 showing discrimination between healthy donors and early-stage tumors; (**B**) ROC curve of miR-339 in combination with miR-140 showing discrimination between healthy donors and late-stage tumors.

The combination of miR-328 and miR-361 increased accuracy, albeit not significantly (*p* = 0.164), in identifying early-stage tumors (AUC ROC 0.84; 95% CI 0.74–0.93) with respect to miR-328 alone (Figure 3A). The relation between miR-328 and miR-361 and the risk of early-stage cancer was analyzed by the fitted logistic regression model and showed an OR of 3.24 (95% CI 1.76–5.94, *p* < 0.0001). Conversely, the combination of miR-339 and miR-140 increased accuracy with respect to miR-339 alone (AUC ROC 0.82, 95% CI 0.70–0.93) in discriminating between healthy donors and advanced-stage tumors. The difference was not, however, significant (*p* = 0.463) (Figure 3B).

### 2.5. Discussion

It has been shown that miRNA expression in peripheral blood is a promising biomarker for the detection of several human tumors [8,10], in particular because of its strong stability against RNase digestion. The majority of published studies have used plasma or serum as the biological starting material and have identified different miRNAs that are up- or downregulated in NSCLC patients with respect to healthy donors [9,17–23]. One of the main problems in using this type of material is the small quantity of miRNAs detectable, which may hinder analysis and the use of the correct housekeeping miRNAs. Several studies have evaluated miRNA expression in peripheral blood mononuclear cells (PBMCs) for its potential as an alternative biological material, showing that specific miRNAs are expressed differently in the PBMCs of healthy donors and in those of individuals with other non-neoplastic lung diseases [11,16]. miRNA expression in peripheral blood has also shown promise as a biomarker in ovarian cancer and mesothelioma [12,14].

The nature of the blood cells that contribute to the miRNA signature is still elusive. The number of circulating tumor cells appears to be too small to strongly influence the miRNA expression pattern in whole blood. It is thought that the tumor influences immune cells through the release of factors such as cytokines. Moreover, previous studies have demonstrated a difference in mRNA signatures between pre-surgery and post-surgery blood samples in lung cancer patients [24], supporting the hypothesis of a communication between the tumor and peripheral blood cells.

We analyzed the expression profiles of a panel of miRNAs in whole peripheral blood of NSCLC patients in relation to the different clinical–pathologic characteristics of patients, and also compared the profiles with those observed in healthy donors. The 14 miRNAs were chosen on the basis of results from two studies [11,16] that compared miRNA expression in whole peripheral blood of NSCLC patients with that of healthy donors or individuals with chronic obstructive pulmonary disease. We selected the miRNAs that showed the highest fold-changes in expression between NSCLC and the other non-cancer groups. We also analyzed the expression of miR-21 and miR-155 as both have been identified as promising biomarkers of NSCLC, albeit evaluated in plasma [21,25]. Expression levels of miR-328, miR-140, miR-18a and miR-339 were significantly higher in the blood cells of NSCLC patients than in those of healthy donors. In particular, miR-328 proved to be the most accurate tumor marker in distinguishing patients with early-stage tumor from healthy donors, with an AUC ROC of 0.82. Arora and co-workers recently reported, for the first time, an association between the presence of miR-328 and lung cancer [26]. In particular, these authors demonstrated an involvement of miR-328 in “brain-seeking” metastatic potential, also showing its overexpression in both primary tumors and metastatic brain lesions. Analysis of mRNA targets of miR-328 revealed the involvement of PRKCA, IL-1beta and c-Raf1, which are all known to be involved in cancer cell migration. We are currently monitoring the patients enrolled in our study to verify whether those with a higher expression of miR-328 have a higher risk of developing brain metastases. In our case series, no significant correlation was observed between miR-328 expression and the presence of brain metastases in late-stage tumors. Conversely, the high diagnostic accuracy of this marker in early-stage tumors would seem to indicate its potential usefulness for the timely identification of malignant nodules.

In the present study miR-339 showed good diagnostic accuracy in distinguishing healthy donors from patients with late-stage tumors. Although this is of little importance for the early diagnosis of NSCLC, it nevertheless indicates the need for a more in depth evaluation of this miRNA in NSCLC.

We also observed a significantly higher expression of miR-423 in patients with SCC than in those with ADC, indicating a specific role of this miRNA in squamous cell carcinogenesis. It has also been shown that miR-423 is overexpressed in head and neck squamous cell carcinomas (HNSCC) and in HNSCC cell lines [27]. Moreover, miR-423 would seem to be involved in the promotion of cell growth and cell cycle progression in hepatocarcinoma cells through the inhibition of p21Cip1/Waf1 [28], and it has also been seen that inhibition of p21 is involved in the neoplastic transformation of oral squamous epithelium [29].

The high stability of miRNAs in human biological fluids renders them ideal biomarkers for the diagnosis of NSCLC. A non-invasive test performed on peripheral blood and capable of discriminating between normal individuals and lung cancer patients could have two potentially important uses: first, it could be implemented as a preliminary screening method to select individuals at high risk of NSCLC who require further investigation with spiral CT, and secondly, it could help to discriminate between neoplastic and non-neoplastic diseases in individuals with suspicious nodules detected by CT scans, thereby avoiding the need for serial CTs or invasive biopsy.

## 3. Experimental Methods

### 3.1. Case Series

A group of 24 healthy donors (9 females, 15 males) recruited by the Blood Transfusion Unit and 86 patients (30 females, 56 males) with histologically or cytologically confirmed NSCLC referred to the Department of Diseases of the Thorax of Morgagni–Pierantoni Hospital in Forlì were enrolled in the study. Neither healthy donors nor patients had a previous history of malignant disease. Median age was 65 years (range 27–87 years) for healthy donors, and 68 (range 45–83 years) for NSCLC patients. Among healthy donors, 11 had never smoked, 7 were former smokers and 6 were current smokers (at least 20 packs of cigarette/year). Among NSCLC patients, 11 had never smoked, 46 were former smokers and 22 were current smokers. This information was missing for the remaining 7 patients.

Sixty-three tumors were adenocarcinomas (ADC), 22 were squamous cell carcinomas (SCC) and 1 was sarcomatoid carcinoma. On the basis of TNM classification 51 tumors were stage I, 3 stage II, 20 stage III, and 12 stage IV. Blood samples were taken after obtaining informed consent from all individuals and prior to any anticancer treatment. The study protocol was reviewed and approved by the local ethics committee.

### 3.2. Sample Collection

Five milliliters of peripheral blood were collected in PAXgene Blood RNA Tubes (Qiagen) specifically designed for the collection and stabilization of cellular RNA from whole blood. Samples were stored at −80 °C until RNA extraction, which was performed within 6 months of collection.

### 3.3. RNA Extraction

RNA was extracted from the blood of controls and patients using TRIzol reagent (Invitrogen). Briefly, PAXgene Blood RNA Tubes were centrifuged at 3000× *g* for 10 min, after which the cell pellets were washed with 4 mL of RNase-free water and re-centrifuged at 3000× *g* for 10 min. The cell pellets were then lysed in TRIzol reagent and RNA extraction was continued as per the manufacturer’s instructions. RNA quantity and quality was assessed with Nanodrop (Thermo Scientific).

### 3.4. miRNA Analysis

Ten nanograms of total RNA were reverse transcribed using TaqMan MicroRNA Reverse Transcription kit (Applied Biosystems, Foster City, CA, USA), according to the manufacturer’s instructions. Expression levels of the different miRNAs were evaluated by Real-Time PCR using TaqMan MicroRNA assays. The Real-Time PCR reactions were performed in triplicate in a total volume of 20 μL. The cycling protocol consisted in an initial step at 95 °C for 10 min followed by 40 cycles each of 15 s at 95 °C and then 60 s at 60 °C. The experiments were performed using the Applied Biosystems ABI7500.

### 3.5. Statistical Analysis

Analysis of the best housekeeping miRNAs was performed using geNorm software, the underlying principles and formulas of which are described by Vandesompele and coworkers [30]. geNorm calculates the gene expression stability measure (M) for a control gene as the average pairwise variation for that gene with all other tested control genes. Stepwise exclusion of the gene with the highest M values permits ranking of the tested genes according to their expression stability.

Nonparametric ranking statistics (median test) were used to analyze the relationship between median values of the different miRNA expression levels and healthy donor and patient characteristics. The most efficient cutoff values to discriminate between healthy donors and cancer patients were identified using receiver operating characteristic (ROC) curve analysis. The true positive rates (sensitivity) were plotted against the false positive rates (1-specificity) for all classification points. 95% confidence intervals (95% CI) were calculated for sensitivity and specificity values. The chi-square test was performed to evaluate differences in sensitivity and specificity between the clinical and smoking habit subgroups. The independent diagnostic relevance of markers considered as continuous variables was analyzed by the logistic regression model in which natural logarithmic concentrations of markers were considered as predictor variables, and cancer status (case/control) was considered as a binary outcome variable [31]. The linear predictor or logit resulting from this multivariable model after stepwise procedure was used as a new diagnostic test for which the ROC curve was calculated. All *p* values were based on two-sided testing and statistical analyses were carried out using SAS Statistical software version 9.1 (SAS Institute).

## 4. Conclusions

In conclusion, we demonstrated that miRNA, especially miR-328 expression in peripheral blood cells, could be a promising biomarker to distinguish between patients with early-stage tumors and healthy donors. A confirmatory study on a larger case series is ongoing to corroborate this potentially important finding.

## Figures and Tables

**Table 1:** AUC ROC values of different miRNAs in the overall case series and in patients with different stages of tumors.

miRNA	AUC ROC (95% CI) Overall case series	AUC ROC (95% CI) Stages I and II	AUC ROC (95% CI) Stages III and IV
miR-328	0.79 (0.70–0.89)	0.82 (0.72–0.92)	0.75 (0.63–0.88)
miR-423	0.70 (0.58–0.82)	0.71 (0.58–0.84)	0.68 (0.54–0.83)
miR-140	0.71 (0.59–0.82)	0.69 (0.56–0.82)	0.74 (0.61–0.87)
miR-18a	0.76 (0.67–0.86)	0.77 (0.66–0.88)	0.76 (0.63–0.89)
miR-339	0.76 (0.66–0.86)	0.74 (0.63–0.86)	0.80 (0.69–0.91)
miR-361	0.62 (0.50–0.74)	0.60 (0.46–0.73)	0.67 (0.53–0.82)

**Table 2:** Sensitivity and specificity of miR-328 in discriminating between early-stage NSCLC and healthy donors.

Cutoff	Sensitivity	95% CI	Specificity	95% CI
250	82	70–90	67	47–83
300	70	58–81	83	66–94
350	63	50–75	83	66–94
400	46	34–60	95	76–99
450	33	22–46	100	-
